# Predicting critical illness mortality and personalizing therapy: moving to multi-dimensional data

**DOI:** 10.1186/s13054-016-1597-6

**Published:** 2017-01-30

**Authors:** Zudin A. Puthucheary, Paul Wischmeyer

**Affiliations:** 1 Institute of Sports and Exercise Health, University College London Hospitals, 1st Floor, 170 Tottenham Court Road, London, W1T 7HA UK; 2 Division of Critical Care, University College London Hospitals, London, UK; 3 Department of Anesthesiology, Duke University of Medicine, Durham, USA; 4 Duke Clinical Research Institute, Duke University of Medicine, Durham, USA

**Keywords:** Skeletal muscle, Intensive care, Mortality

Predicting mortality has been a corner piece of critical care research and practise dating back to the first descriptions of the Acute Physiology and Chronic Health Evaluation score in 1981 [1]. With an increasing burden of non-communicable disease in modern society, pre-existing functional status seems to an important contributor to outcome prediction. Poor physical function is an important predictor of mortality in ambulant diseases. The construct of frailty [2], translated from older patient care into critical care, has provided a useful language to discuss pre-morbid functional status.

Whilst frailty can be established from history taking or hospital coding, both of these methods have clinical and research methodological disadvantages. Wilhelmus et al. [3] now offer an alternative approach in a retrospective analysis of computerized tomography scans. Muscle quality as defined by Hounsfield units at the level of L3 was investigated in 491 patients, with a threshold set to define intramuscular adipose tissue and visceral adipose tissue. Higher skeletal muscle density (i.e. better quality) was associated with lower 6-month mortality and shorter hospital length of stay after correction for muscle mass and severity of illness.

Skeletal muscle quality is recognized as a marker of function in healthy individuals [4] and critically ill patients [5]. Alterations are seen with aging [4], immobilization [6], chronic disease states [7] and critical illness [8]. These conditions demonstrate qualitative changes in muscle structure as a result of increasing collagen and lipid deposition [6]. Intramyocellular lipid accumulation is additionally a hallmark of metabolic diseases, and may exacerbate tissue metabolic derangements in the critically ill.

A limitation of these data is the inability to relate either chronic disease states and poor muscle quality, or muscle quality and functional outcomes. However, decreased skeletal muscle density as an independent predictor of mortality raises the possibility of its use in multi-dimensional scoring systems such as the NUTRIC score [9] or as an alternative marker of chronic poor physiological reserve in the APACHE system.

A number of key roles for early trajectory assessments exist. First, novel early outcome predictors are needed to guide patient and family expectations and decision-making. These need to not only predict risk of death, but also disability, so a patient’s wishes may be honoured and a realistic appraisal of functional outcomes can be made. It is vital we improve upon our ability to inform patients and families early in critical illness on the likelihood of significant morbidity. It is possible that admission skeletal muscle quality and quantity may be key to this discussion in the future. Ongoing testing via lean body mass ultrasound [8] and other modalities [10] may also be vital to continued discussions of prognosis. Second, these tests of muscle quality should assist in guiding therapy. A recent post-ICU recovery consensus conference indicated that a major gap exists in understanding how to effectively and efficiently screen patients for specific post-ICU impairments to determine the need for further diagnostic work-up and treatment [11]. Thirdly, the current controversy around personalizing nutrition delivery in the ICU to optimize outcome [12] has begun to be addressed by early studies validating the role of the aforementioned NUTRIC score in nutrition risk prediction [9]. High malnutrition risk patients may benefit to a greater degree than those with lower risk. A key addition to this prediction of nutrition risk may be muscle quantity and quality at ICU admission. The ability of the muscle to utilize substrate such as lipid and overall glycogen content [10] may be key in delivering personalized nutrition to improve outcomes. Patients with low muscle quality and quantity may have greater and different specific nutritional requirements. Conversely, increased muscle myosteatosis as defined by decreased skeletal muscle density or increased intermuscular adipose tissue may indicate impaired muscle substrate utilization as implied by Wilhelmus et al. [3]. This may indicate that nutrition delivery needs to account for impaired substrate (lipid) utilization and/or measures need to be taken to improve muscle lipid uptake/utilization (e.g. carnitine [13]). Finally, exercise and reduction of immobility are essential to reduce impaired muscle substrate metabolism and thus improve poor muscle quality.

These assessments may be a key innovation prior to major surgery or cancer therapy. Patients with poor skeletal muscle quality could then be enrolled in prehabilitative exercise/nutrition programmes to improve skeletal muscle quality and quantity [14]. Clinical trials systematically evaluating muscle quality and quantity measures via CT scan and ultrasound could then be performed to assess interventions and target ideal methods to optimize patients. Further, in the ICU, these techniques need further study to determine the muscle-level effects of individual nutrition (e.g. protein delivery, anabolic agents [10]) and specific ICU-rehabilitation interventions (e.g. in-bed ergometry, functional electrical stimulation [15]). Current functional testing (i.e. Medical Research Council sum score, hand-grip strength, walk testing) is both volitional and not muscle specific, and has significant implementation, interpretation and compliance challenges. Thus, the role of muscle quality and quantity measurement described here deserves additional study and validation to add an additional “dimension” to our prediction of outcome and personalization of care in the ICU.

## Abbreviations

APACHE: Acute Physiology and Chronic Health Evaluation
CT: Computerized Tomography
ICU: Intensive care unit
NUTRIC: Nutrition Risk in Critically Ill
